# Awareness and Acceptance of Digital Rectal Examination for the Clinical Evaluation of Anorectal Conditions Among the Saudi Population: A Cross-Sectional Study

**DOI:** 10.7759/cureus.41873

**Published:** 2023-07-14

**Authors:** Jubran J Al Faifi, Musaab M AlAradi, Naif A Alomar, Farah F AlMuqrin, Reem M AlKublan

**Affiliations:** 1 General Surgery, College of Medicine, Imam Mohammad Ibn Saud Islamic University, Riyadh, SAU; 2 Medicine and Surgery, College of Medicine, Imam Mohammad Ibn Saud Islamic University, Riyadh, SAU

**Keywords:** evaluation, anorectal, digital rectal examination, acceptance, awareness

## Abstract

Introduction: Digital rectal examination (DRE) is an important diagnostic tool used by physicians to resolve several confusing clinical situations. The history and physical examination cannot be complete without performing a DRE. Any patient that presents with abdominal complaints (e.g., diarrhea, constipation, nausea, vomiting, abdominal or rectal pain, bleeding) needs a DRE which is important for detecting warning signs of serious conditions that require further investigation and evaluation such as malignancies. Therefore, our aim was to assess and measure the awareness of the Saudi population regarding the importance and acceptance to perform DRE.

Methods: This cross-sectional study was conducted in Riyadh, the capital city of Saudi Arabia, using an online survey between September 2022 and March 2023; the targeted participants were adults between the ages of 18 to 75.

Results: The study indicated that the general community awareness of DRE is low, with only 59.1% of participants having heard of DRE and 14.6% having undergone the procedure previously. The majority of individuals (60.9%) were willing to undergo DRE if a healthcare provider suggested it. Participants' knowledge of DRE's ability to detect various anorectal diseases varied. While the majority of individuals believed DRE could detect hemorrhoids, just 40.4% believed DRE could help detect colorectal cancer. Chronic constipation or diarrhea, feces-induced stretching, and prolonged sitting were the most oft-cited causes of hemorrhoids. Anemia was the most often reported consequence of hemorrhoids, followed by hypertension and diabetes.

Conclusion: The significance of DRE as a screening tool for the early detection and prevention of anorectal problems, as well as the need for adequate care and treatment of hemorrhoids to prevent complications, are highlighted by these findings. Healthcare practitioners should actively recommend and provide information about DRE and other screening technologies, as well as address their patients' concerns and misconceptions.

## Introduction

Digital rectal examination (DRE) comprises simultaneous visual inspection of the perianal skin, manual palpation of the rectum, and assessment of the perineal neuromuscular function [1] and constitutes an important diagnostic tool that is clinically used by physicians for differential diagnosis [2]. History-taking and physical examination cannot be completed without a DRE as any patient who presents with abdominal complaints such as diarrhea, constipation, nausea, vomiting, abdominal or rectal pain, or rectal bleeding, needs a rectal examination; this is important for detecting findings indicative of serious conditions such as malignancies that require further investigation and evaluation [3]. The age at which DRE is required and the frequency of DRE remains debatable. The American Cancer Society recommends that DRE and stool examination with occult blood testing should be performed annually for all individuals aged 40 or more to screen for both prostate and colorectal cancer [4].

An inspection of the buttocks of the patients can provide clues to many disorders including hemorrhoids, skin tags, fissures, and fistulous tracts in patients with inflammatory bowel disease, rectal prolapse, polyps, tumors, and ulcers caused by herpes simplex or syphilis [5]. The perianal skin may be affected by dermatological conditions including psoriasis and vitiligo, or infections such as syphilitic dermatitis and candidiasis. The assessment of neuromuscular function using DRE is unavoidable in conditions such as the cauda equina syndrome or multiple sclerosis wherein loss of neuromuscular function can cause fecal incontinence, which may constitute one of the first symptoms of serious systemic diseases, including neuropathies and spinal cord-compressing masses [6-8].

There are contraindications to performing a DRE which are as follows: immunocompromised patients (the risk of introducing infection can be potentially life-threatening), the absence of anus, imperforate anus, prolapsed thrombosed internal hemorrhoids, stricture, severe anal pain, and/or an unwilling patient.

Therefore, the importance and relevance of DRE should not be underestimated as DRE can prove lifesaving through early detection of serious diseases. This study was conducted to assess and measure the awareness and acceptance of the Saudi population regarding the importance of DRE.

## Materials and methods

This cross-sectional study involved a survey conducted between September 2022 and March 2023 in Riyadh, the capital of Saudi Arabia. The study population comprised Adult Saudi residents of Riyadh aged between 18 to 75 years who consented to participate in this research. The study was approved by the Institutional Review Board of Al-Imam Mohammad Ibn Saud Islamic University (approval no: HAPO-01-R-001).

Data were acquired using a bilingual (Arabic and English) self-administered Google form online questionnaire that comprised 23 questions pertaining to demographic variables, awareness and acceptance of DRE, and knowledge of hemorrhoids (Appendices). The demographic component of the questionnaire included questions on age, sex, nationality, level of education, employment status, field of employment (medical or non-medical), and history of anorectal disorders. The DRE awareness and acceptance section of the questionnaire portion asked participants if they had heard of DRE and had ever undergone a DRE. Participants were asked if they would consent to a DRE and why they would or would not. The section on hemorrhoids contained questions on its definition, etiology, consequences, and prevention.

Prior to the initiation of the main investigation, a pilot study with 20 participants was undertaken to test the validity and reliability of the questionnaire and revealed a Cronbach's alpha coefficient of 0.707, which is acceptable. The results of the pilot study helped refine the wording and format of the questionnaire to ensure clarity and readability. Before answering the questionnaire, all participants provided informed consent, and the data collected were utilized only for research purposes while ensuring anonymity. The anticipated response rate was between 300 and 600. Individuals aged 18 to 75 years who agreed to participate in the study were included, whereas those who declined or were outside the stipulated age range were excluded.

The acquired data were analyzed using SPSS version 25 (IBM Corp., Armonk, NY). Descriptive statistics were utilized to describe the demographic features of the participants, and a chi-square test was utilized to investigate the relationship between demographic characteristics and knowledge and acceptance of DRE.

## Results

In total, there were 406 respondents to the survey, but only 384 of them were suitable for this study; the other 22 responses were excluded because they were not from Saudi Arabia.

The demographic characteristics of the participants are shown in Table 1. The majority of participants (64.8%) were female, with an age range of 18 to 65 years. The largest proportion of participants were in the 18 to 25 years age group (46.4%). The majority of participants were university-educated (80.2%) and either unemployed (60.4%) or students (36.2%); 66.9% of participants were from non-medical sectors and 28.4% of individuals indicated a personal or familial history of anorectal disorders.

**Table 1:** Demographic factors of the participants (N=384)

		Count	Column N %
Sex	Male	135	35.2%
	Female	249	64.8%
Age,years	18-25	178	46.4%
	26-45	116	30.2%
	46-65	70	18.2%
	>65	20	5.2%
Educational level	Primary	1	0.3%
	Middle	7	1.8%
	Secondary	68	17.7%
	University	308	80.2%
Employment status	Employed	152	39.6%
	Not employed	93	24.2%
	Student	139	36.2%
Work field	Medical	127	33.1%
	Non-medical	257	66.9%
Have you had a history of anorectal conditions?	No	278	72.4%
	Yes, me	71	18.5%
	Yes, relative	35	9.1%

Table 2 displays the participants' knowledge and attitudes with regard to DRE. Approximately 59.1% of participants had heard of DRE, but only 14.6% had undergone DRE; 60.9% of participants indicated that they would undergo a DRE if a healthcare physician advised it. Only 12.5% of interviewees felt that the DRE is excessively intrusive and should not be used by physicians. When asked whether customs or traditions pose a societal barrier in postponing or not going to the doctor for a DRE, 64.0% of participants responded "yes" whereas only 12.0% said "no" and 27.6% responded "maybe." With regard to the use of folk medicine to treat anorectal diseases, 68.8% of participants said they would not avail of such therapy whereas 9.1% reported that they would and 22.1% responded "maybe." Only 39.8% of participants said that they would consent to be examined in screening clinics for anorectal diseases that employ DRE; whereas, 35.2% said they would not and 25.0% responded "maybe." When asked if they would see a doctor promptly if they or a family member were diagnosed with anorectal diseases, 76.2% of participants responded "yes", only 4.8% responded "no", and 19.0% responded "maybe."

**Table 2:** Awareness and attitude toward digital rectal examination DRE, digital rectal examination

		Count	Column N %
Have you ever heard of DRE?	Yes	227	59.1%
	No	157	40.9%
Have you ever had a DRE performed?	Yes	56	14.6%
	No	328	85.4%
If your doctor recommended performing a DRE, would you accept?	Yes	234	60.9%
	No	150	39.1%
Do you think that DRE is too invasive and should not be used by doctors?	Yes	48	12.5%
	No	245	63.8%
	I do not know	91	23.7%
Do you think customs or traditions constitute a societal barrier in delaying or not going to the doctor for DRE?	Yes	232	60.4%
	No	46	12.0%
	Maybe	106	27.6%
If you or one of your family members were injured, would you resort to folk medicine?	Yes	35	9.1%
	No	264	68.8%
	Maybe	85	22.1%
If there were clinics that use DRE to screen for anorectal conditions, would you agree to be examined?	Yes	153	39.8%
	No	135	35.2%
	Maybe	96	25.0%

**Figure 1:**
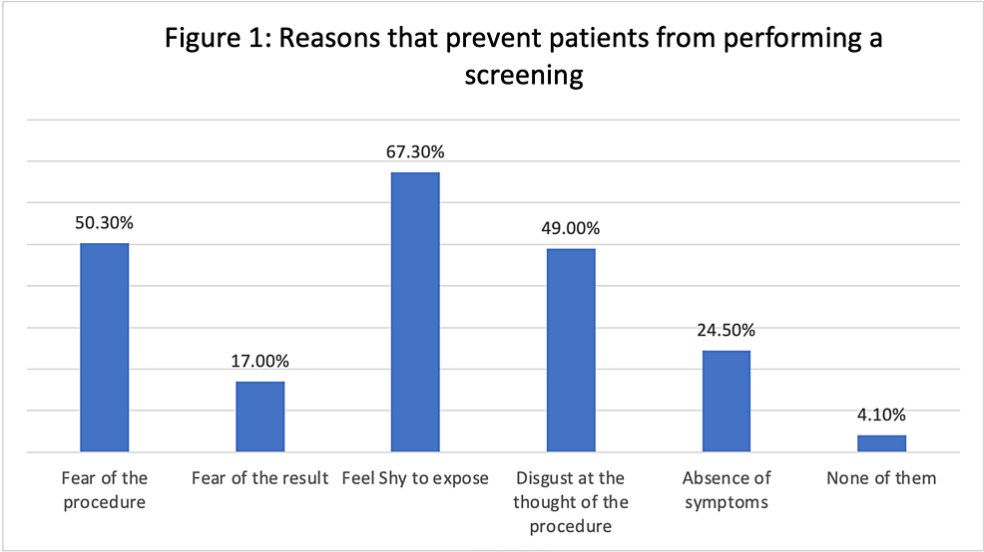
Reasons that prevent patients from performing a screening

As shown in Figure 1, the most common factors that stopped patients from undergoing a test were a reluctance to expose themselves (67.3%) and a fear of the procedure (50.3%). As reported by 49.0% of participants, disgust at the thought of the procedure was a significant issue. Fear about the result and absence of symptoms were less common, as reported by 17.0% and 24.5% of the participants, respectively; 4.1% of patients said that none of the aforementioned reasons prohibited them from undergoing a DRE-based screening.

Table 3 displays the participants' perceptions of symptoms and diseases requiring DRE. The majority of participants (63.3%) said that they would consent to a DRE in the event of pain, whereas bleeding (56%) and lumps (55%) were frequently reported symptoms that would prompt participants to consent to a DRE. A small proportion of participants (20.8%) claimed that a change in bowel habits would make them accept DRE, whereas 12.2% reported that nothing would make them accept a DRE. With regard to the participants' perceptions of the DRE's utility for identifying various conditions, the majority of respondents (64.8%) believed that the DRE could detect hemorrhoids and the general belief was that colorectal cancer may be detected by DRE, with 40.4% of participants reporting this assumption. Anal skin tags (29.2%), anal fissures (33.9%), and prostate cancer (28.4%) were all conditions that DRE might detect. Approximately 21.9% of individuals did not know which conditions may be detected with DRE.

**Table 3:** The perception of the participants toward symptoms and conditions that required DRE DRE, digital rectal examination

		Frequency	Percent
Which symptoms would make you accept the DRE?	Bleeding	215	56.0%
	Pain	243	63.3%
	Lumps	212	55.2%
	Change in bowel habits	80	20.8%
	Nothing	47	12.2%
Which of these conditions do you think a DRE would help detect?	Hemorrhoids	249	64.8%
	Colorectal cancer	155	40.4%
	Anal skin tags	112	29.2%
	Anal fissure	130	33.9%
	Prostate cancer	109	28.4%
	Don’t know	84	21.9%

**Figure 2:**
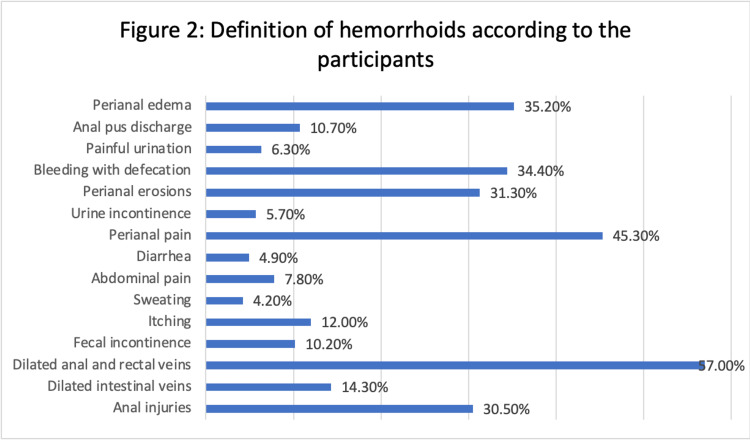
Definition of hemorrhoids according to the participants

The participants were allowed to choose more than one choice as a definition of hemorrhoids (Figure 2). The most popular definition, selected by 57.0% (219) of respondents was "dilated anal and rectal veins". Perianal pain (45.3%) (174), perianal erosions (31.3%) (120), and defecation-related bleeding (34.4%) (132) were also frequently associated with hemorrhoids. Anal injuries (30.5%) (117), itching (12%) (46), perianal adenoma (35.2%) (135), anal pus discharge (10.7%) (41), fecal incontinence (10.2%) (39), sweating (4.2%) (16), abdominal pain (7.8%) (30), diarrhea (4.9%) (19), painful urination (6.3%) (24), and urinary incontinence (6.3%) (24) were additional symptoms that participants associated with hemorrhoids (5.7%) (22).

Table 4 displays the summary of the participants' knowledge of hemorrhoids’ causes, consequences, and preventative treatments. The most oft-cited causes of hemorrhoids were chronic constipation or diarrhea (63.0%), defecation-related stretching (58.6%), and extended periods of sitting (44.5%). Pregnancy (24.5%) and labor (31.0%) were also commonly cited as contributory factors. Consuming an excessive amount of vegetables (4.9%) and oral contraceptive use (3.1%) were the least reported causes. Anemia was the most frequent complication of hemorrhoids (67.4%), followed by hypertension (38.6%) and diabetes mellitus (14.4%). The most oft-mentioned preventative approach against hemorrhoids was the consumption of fiber-rich foods (77.8%), followed by excessive fluid intake (72.8%) and defecation on demand (53.3%). Other preventative strategies included quitting smoking (15.1%), avoiding fatty foods (29.0%), using tissues for cleaning (12.8%), drinking hot beverages (10.2%), fasting (8.9%), and getting enough sleep (12.8%).

**Table 4:** Knowledge of the participants about the causes, complications, and preventive measures toward hemorrhoids

		Frequency	Percent
Causes of hemorrhoids	Using the bathroom for long periods of time	139	36.2%
	Eating too many vegetables	19	4.9%
	Long-duration sitting	171	44.5%
	Hot bathing with Jacuzzi	31	8.1%
	Defecation stretching	225	58.6%
	Tight pants	24	6.3%
	No sports	46	12.0%
	Chronic constipation or diarrhea	242	63.0%
	Oral contraceptive pill	12	3.1%
	Pregnancy	94	24.5%
	Labor	119	31.0%
Complications of hemorrhoids	Hypertension	148	38.6%
	Diabetes mellitus	55	14.4%
	Anemia	258	67.4%
Preventive measures to prevent hemorrhoids	Sufficient sleeping hours	49	12.8%
	Stop smoking	58	15.1%
	Eating fiber-rich food	298	77.8%
	Avoid fatty food	111	29.0%
	Hot drinks	39	10.2%
	Defecation on need	204	53.3%
	Fasting	34	8.9%
	Use tissue for cleaning	49	12.8%
	Excessive fluid intake	279	72.8%

Table 5 illustrates the relationship between participants' attitudes, awareness of DRE, and demographic characteristics. The majority of individuals (57.3%) indicated that they would consent to a DRE if their physician advised it. Those who had heard about DRE were more inclined to accept a DRE if their doctor advised one (P = 0.001). Similarly, participants with a previous history of anorectal problems were more likely to have heard of DRE (p <0.001) and to accept a DRE if it was prescribed by a physician (p = 0.012). Regarding demographic characteristics, sex, and work status were strongly linked with physician-recommended DRE acceptance (p = 0.003 and 0.008, respectively). Male participants are more likely than female participants to accept DRE if prescribed by their doctor. Employed participants were more likely than unemployed or student participants to embrace DRE if suggested by their doctor. Age and educational level were related to DRE acceptance, but not for all age groups or educational levels. Participants between ages 46 and 65 and those with a college education were more likely to accept DRE if advised by their physician. The employment sector was not significantly connected with DRE acceptability.

**Table 5:** The relation between attitude, awareness of the participants toward digital rectal examination and demographic factors of the participants DRE, digital rectal examination

		Have you ever heard of DRE?					If your doctor recommended performing a DRE, would you accept?				
		Yes		No		P-value	Yes		No		P-value
		Count	Row N %	Count	Row N %		Count	Row N %	Count	Row N %	
Gender	Male	94	69.6%	41	30.4%	0.002*	96	71.1%	39	28.9%	0.003*
	Female	133	53.4%	116	46.6%		138	55.4%	111	44.6%	
Age	18-25	111	62.4%	67	37.6%	0.560	100	56.2%	78	43.8%	0.026*
	26-45	68	58.6%	48	41.4%		68	58.6%	48	41.4%	
	46-65	37	52.9%	33	47.1%		49	70.0%	21	30.0%	
	<65	11	55.0%	9	45.0%		17	85.0%	3	15.0%	
Educational level	Primary	1	100.0%	0	0.0%	0.036*	1	100.0%	0	0.0%	0.408
	Middle	6	85.7%	1	14.3%		4	57.1%	3	42.9%	
	Secondary	31	45.6%	37	54.4%		36	52.9%	32	47.1%	
	University	189	61.4%	119	38.6%		193	62.7%	115	37.3%	
Employment status	Employed	91	59.9%	61	40.1%	0.119	107	70.4%	45	29.6%	0.008*
	Not employed	47	50.5%	46	49.5%		53	57.0%	40	43.0%	
	Student	89	64.0%	50	36.0%		74	53.2%	65	46.8%	
Work field	Medical	99	78.0%	28	22.0%	0.000*	74	58.3%	53	41.7%	0.451
	Non-medical	128	49.8%	129	50.2%		160	62.3%	97	37.7%	
Do you have a history of anorectal conditions?	No	147	52.9%	131	47.1%	0.000*	157	56.5%	121	43.5%	0.012*
	Yes, me	52	73.2%	19	26.8%		50	70.4%	21	29.6%	
	Yes, relative	28	80.0%	7	20.0%		27	77.1%	8	22.9%	

## Discussion

This study examined the participants' knowledge, attitudes, and perceptions of DRE and anorectal diseases such as hemorrhoids. According to our findings, only 59.1% of participants in the current study had heard of the DRE, and 14.6% had already received it, which aligns with results from earlier studies. Only 78% of males would participate in a test that included both a DRE and a prostate-specific antigen, demonstrating a lack of understanding or interest in DRE [9]. Similar findings were made by Lee et al., who found that 24% of people reported having annual DREs and 33% had never had one, indicating a need for increased DRE awareness and use [10]. However, most participants (60.9%) claimed they would consent to DRE if a medical professional recommended it. These data suggest that healthcare professionals should increase public awareness regarding DRE.

Furthermore, DRE prevalence was 41.6% in men who used the publicly funded healthcare system and 63.3% in those who used services associated with private health insurance [11]. These data imply that access to healthcare services may influence DRE adoption. By increasing knowledge, education, and access to healthcare services, DRE utilization can be increased, resulting in earlier detection and prevention of anorectal disorders. Healthcare providers should play an active role in suggesting and disseminating information on DRE, particularly among groups with lower rates of knowledge and utilization. Additionally, efforts should be made to expand access to healthcare services, particularly among underprivileged communities, such that all individuals can undergo DRE and receive appropriate therapy.

With 60.4% of participants answering "yes" to this question, the survey also demonstrated that customs and traditions might hinder societal postponement or avoidance of DRE-related treatment. This result is comparable to the findings of previous studies which indicated that traditions could be seen as significant hurdles to DRE [12,13]. In addition, reluctance to expose themselves (67.3%) and dread of the process were the most prevalent factors stopping patients from undergoing screening (50.3%). In an earlier study, the authors established that patients' rejection of DRE during prostate cancer screening is primarily due to the absence of lower urinary tract symptoms, misconceptions about prostate cancer screening, and embarrassment, particularly when screening for the first time. The most common reasons given for not getting screened were the fear of learning something was wrong (48.1%), not knowing what would be done during the screening (54.3%), believing that prostate cancer is not a severe condition (55.8%), and thinking that rectal examination is embarrassing (56.6%), according to a previous study done in Oman [14]. These data show that, in order to educate patients about the value of DRE and early detection of anorectal illnesses, healthcare professionals must overcome social and cultural barriers.

When asked about their perceptions of symptoms and conditions requiring DRE, participants generally stated that they would accept the examination in the case of pain (63.3%). Participants frequently mentioned bleeding (56%) and lumps (55%) as symptoms that would lead them to accept DRE, and this is similar to what was reported in several studies, showing that the presence or absence of symptoms would affect a patient's decision to undergo DRE [15,16]. The abovementioned results imply that patients may link DRE with the existence of symptoms; therefore, healthcare professionals should emphasize the relevance of DRE as a screening tool for early identification and prevention of anorectal diseases.

The study additionally demonstrated that participants' understanding of DRE's utility for detecting diverse pathological scenarios varied. Only 40.4% of people thought DRE could help detect colorectal cancer, compared to 64.8% who believed it could help diagnose hemorrhoids. DRE is an effective method for detecting and preventing anorectal diseases, including colorectal cancer [17,18] which is the second most common disease and the second major cause of cancer-related mortality worldwide [19,20]. People with an average risk of colorectal cancer should begin screening at age 45, according to the American Cancer Society. Besides colorectal cancer, DRE can be utilized to detect anorectal conditions such as hemorrhoids [21]. Hemorrhoids are a frequent ailment marked by enlarged and irritated veins in the anus and rectum [22,23]. DRE can assist in identifying the presence of hemorrhoids, guide therapy, and help prevent problems. These results emphasize the significance of patient education on the DRE's potential to detect various anorectal disorders.

Hemorrhoids are frequently attributed to chronic constipation, diarrhea, feces-induced stretching, and prolonged sitting [24]. Hemorrhoids are brought on by inflamed and swollen veins in the anal and rectal regions which can lead to discomfort, bleeding, and other symptoms [25]. Chronic diarrhea or constipation can induce strain with bowel movements, increasing the pressure in the anal and rectal areas and causing hemorrhoids. Similar to that of holding or stretching out the tract by the feces during extended defecation, hemorrhoids can develop due to straining and increased pressure. Furthermore, prolonged sitting on a hard surface may lead to increased pressure in the anal and rectal regions that could lead to the formation of hemorrhoids due to prolapse of the veins due to increased anorectal pressure.

The most frequent side effect of hemorrhoids was anemia (67.4%), followed by hypertension (38.6%), and diabetes mellitus (14.4%). Another study reported that the most common side effect of hemorrhoids was anemia, which is consistent with other earlier studies [26,27]. Bleeding from hemorrhoids can result in blood loss and anemia which is characterized by an insufficiency of red blood cells and can cause fatigue and other symptoms [28]. This study identified hypertension and diabetes as potential consequences of hemorrhoids. Several factors, including weight, stress, and inactivity, can contribute to hypertension [29]. Hemorrhoids can cause discomfort and agony, resulting in stress and reduced physical activity, possibly contributing to hypertension. Insufficient insulin synthesis or inefficient insulin usage are the hallmarks of diabetes mellitus which is a metabolic disorder [30]. As constipation and other factors might increase pressure in the anal and rectal regions, hemorrhoids may be more common in patients with diabetes due to constipation. These findings imply that patients may have misconceptions regarding the origins and complications of hemorrhoids; therefore, healthcare practitioners should provide appropriate information and patient education regarding these disorders.

Patients may have some understanding of hemorrhoid prevention. Nevertheless, healthcare providers should emphasize the importance of adopting a healthy lifestyle which includes regular exercise, proper water, and a fiber-rich diet. The most oft-suggested preventative methods for hemorrhoids were consuming fiber-rich foods (77.8%), excessive fluid consumption (72.8%), and defecating when necessary (53.3%). As additional preventive measures, smoking cessation and avoidance of extended sitting or standing should be emphasized.

Finally, this study explored the relationship between participants' attitudes, their awareness of DRE, and their demographic characteristics. Participants who had previously experienced anorectal problems were more likely to have heard of DRE and to agree to DRE if recommended by a physician. The likelihood that a person would accept DRE if offered by a doctor was significantly influenced by their sex and work status, with male and employed individuals being more inclined to accept DRE. These findings imply that healthcare professionals should consider patient demographics such as sex and work position when recommending DRE and educating patients about anorectal problems.

The were some limitations to the study; there was trouble in the process of data collection and the time was prolonged due to the refusal of some people to participate in the questionnaire due to its title. Therefore, we assigned data collectors. Our recommendations for further research include expanding the study sample for a more precise representation, shifting to a paper-distributed questionnaire to reduce bias, and involving participants from more regions of Saudi.

## Conclusions

In conclusion, the current study emphasizes the necessity for patient education and knowledge of the significance of DRE as a screening tool for early detection and prevention of anorectal diseases. The results of the study provided valuable insights into the level of awareness and acceptance of DRE among adults in Riyadh, Saudi Arabia, and could assist healthcare professionals in developing strategies to promote the importance of DRE screening and increase the general population's acceptance of this clinical examination. Healthcare providers should address cultural and societal barriers and provide correct information about the causes, symptoms, consequences, and prevention of anorectal disorders. In addition, healthcare practitioners should consider patient demographics when prescribing DRE and educate patients to help promote acceptance and utilization of this essential screening technique.

## Appendices

Questionnaire for: Digital Rectal Examination: The Awareness Level and Acceptance among Saudi Population for the Clinical Evaluation of Anorectal Conditions

All the information gathered is for research purposes only

1. Do you agree to participate in this study? Yes No

2. Gender?

Male

Female

3. Age?

• 18-25

• 26-45

• 46-65

• <65

4. Nationality?

Saudi Non Saudi

5. Education level?

Primary Middle Secondary

University

6. Employment status ?

Employed Not employed Student

7. Work field?

Medical

Non-medical

8. Do you have a history of anorectal conditions ?

No Yes, me Yes, relative

9. Have you ever heard of digital rectal examination?

Yes No

10. Have you ever performed a digital rectal examination?

Yes No

12. If your doctor recommended performing a digital rectal examination, would you accept? Yes No

13. which of the following could prevent you from performing a screening? (more than one answer is acceptable)? Fear of the procedure Fear of the result

Feel Shy to expose

Disgust at the thought of the procedure

Absence of symptoms

None of them

14. What symptoms would make you accept performing the examination? (more than one answer is acceptable)

Bleeding Pain

Lumps Constipation Change in bowel habits

Nothing

15. Do you think that digital rectal examination would detect which of the following? (more than one answer is acceptable)

Hemorrhoids

Colorectal cancer

Anal skin tags

Anal fissure

Prostrate cancer

Don’t know

16 .Do you think that digital rectal examination is too invasive and should not be used by doctors ? Yes No

I don’t know

17. What is the definition of Hemorrhoids? (more than one answer is acceptable) Anal injuries Dilated intestinal veins

Dilated anal and rectal veins Fecal incontinence Itching Sweating

Abdominal pain Diarrhea Perianal pain Urine incontinence

Perianal errosions

Bleeding with defecation

Painful urination

Anal pus discharge

Perianal edema

18. What are the causes of Hemorrhoids? (more than one answer is acceptable) Long standing in bathroom Eating too much vegetables

Long duration setting Hot bathing with Jacuzzi Defecation stretching The Frankish Chair Tight pants No sports Chronic constipation or diarrhea Oral contraceptive pill Pregnancy Labor

19. What do you think the complications of Hemorrhoids? (more than one answer is acceptable) Hypertension Diabetes mellitus

Anemia

20. What do you think the preventive measures are? (more than one answer is acceptable) Sufficient sleeping hours Stop smoking

Eating fiber-rich food Avoid fatty food Hot drinks Defecation on need Fasting

Use tissue for cleaning

Excessive fluid intake

22. Do you think customs or traditions constitute a barrier for society in delaying or not going to the doctor for digital rectal examination ? Yes No

Maybe

23. If you or one of your family members were injured, would you resort to folk medicine? Yes No

Maybe
